# PomBase 2018: user-driven reimplementation of the fission yeast database provides rapid and intuitive access to diverse, interconnected information

**DOI:** 10.1093/nar/gky961

**Published:** 2018-10-13

**Authors:** Antonia Lock, Kim Rutherford, Midori A Harris, Jacqueline Hayles, Stephen G Oliver, Jürg Bähler, Valerie Wood

**Affiliations:** 1 Department of Genetics, Evolution and Environment, University College London, London WC1E 6BT, UK; 2 Cambridge Systems Biology Centre and Department of Biochemistry, University of Cambridge, Cambridge CB2 1GA, UK; 3 Cell Cycle Laboratory, The Francis Crick Institute, London NW1 1AT, UK

## Abstract

PomBase (www.pombase.org), the model organism database for the fission yeast *Schizosaccharomyces pombe*, has undergone a complete redevelopment, resulting in a more fully integrated, better-performing service. The new infrastructure supports daily data updates as well as fast, efficient querying and smoother navigation within and between pages. New pages for publications and genotypes provide routes to all data curated from a single source and to all phenotypes associated with a specific genotype, respectively. For ontology-based annotations, improved displays balance comprehensive data coverage with ease of use. The default view now uses ontology structure to provide a concise, non-redundant summary that can be expanded to reveal underlying details and metadata. The phenotype annotation display also offers filtering options to allow users to focus on specific areas of interest. An instance of the JBrowse genome browser has been integrated, facilitating loading of and intuitive access to, genome-scale datasets. Taken together, the new data and pages, along with improvements in annotation display and querying, allow users to probe connections among different types of data to form a comprehensive view of fission yeast biology. The new PomBase implementation also provides a rich set of modular, reusable tools that can be deployed to create new, or enhance existing, organism-specific databases.

## INTRODUCTION

The fission yeast *Schizosaccharomyces pombe* is an established model organism for the study of conserved eukaryotic cellular processes. Over the past 70 years, fission yeast research has significantly contributed to many aspects of cell biology, including mechanisms of cell-cycle control, chromosome dynamics and epigenetics (1). In addition, it is increasingly used as a pharmacological and biotechnological tool (2–5).

Since its launch in 2012, PomBase (www.pombase.org) (6,7), the model organism database (MOD) for *S. pombe*, has driven innovation in the MOD community. Like all MODs, PomBase is committed to the timely, comprehensive representation of all data from literature and bioinformatics resources pertaining to its subject species, in formats suitable for human use and computational analysis (8).

With ∼5000 unique visitors per month (verified by Google Analytics), PomBase serves a community considerably larger than the core of about 2000 dedicated fission yeast researchers, reflecting the importance of *S. pombe* as a model for conserved biological phenomena. Moreover, PomBase’s manually curated data corpus provides one of the richest sources of experimental functional annotations currently available for transfer to orthologs in other species.

The primary aims of PomBase are:

- To support day-to-day activities of fission yeast researchers, such as generating hypotheses, planning experiments, and analyzing and interpreting data;
- To integrate manual gene curation into network-based displays and pathway models;
- To promote and support the use of fission yeast as a model eukaryotic system, emphasizing relevance to human biology (∼70% of fission yeast proteins have identifiable human orthologs);
- To provide a community hub that enables fission yeast researchers to interact, and to contribute in-depth curation based on their work.

In October 2017, we released a new version of PomBase, in which both user-facing displays and tools, and underlying technology, have been thoroughly overhauled.

New pipelines eliminate redundancy in data storage and release, and replace time-consuming manual procedures with automated data integrity checks. These changes have improved website stability, made daily data updates feasible and freed both computational resources and staff time to expedite the development and release of new features.

Priorities for new features are guided by our observation that the amount of data produced in scientific laboratories is increasing at a non-linear rate (9), creating a need for intuitive tools and displays to make sense of the ever more rapidly accumulating information. Accordingly, new pages and enhancements of existing pages offer alternate routes to finding information. For example, in addition to gene-centric pages, curated data can now be retrieved by ontology classification, publication, and, for phenotypes, by genotype. On all pages, annotation displays make creative use of ontology structure to support both non-redundant summary views and comprehensive detailed views of curated data.

We have also updated the PomBase home page to highlight recent fission yeast research and community curation, and deployed the JBrowse genome browser to provide intuitive access to genome-scale datasets.

## DATA INTEGRATION AND VISUALIZATION

Gene pages organize detailed information for a range of data types, including Gene Ontology (GO) annotation (10,11), phenotypes, interactions, expression data and sequence information, thereby associating many concepts with one gene. To make this large and complex body of data meaningful, PomBase has introduced features that facilitate access to curated data and that build links between annotations that reflect connections in the biological systems they represent.

### A system-wide biological summary

**Figure 1.**
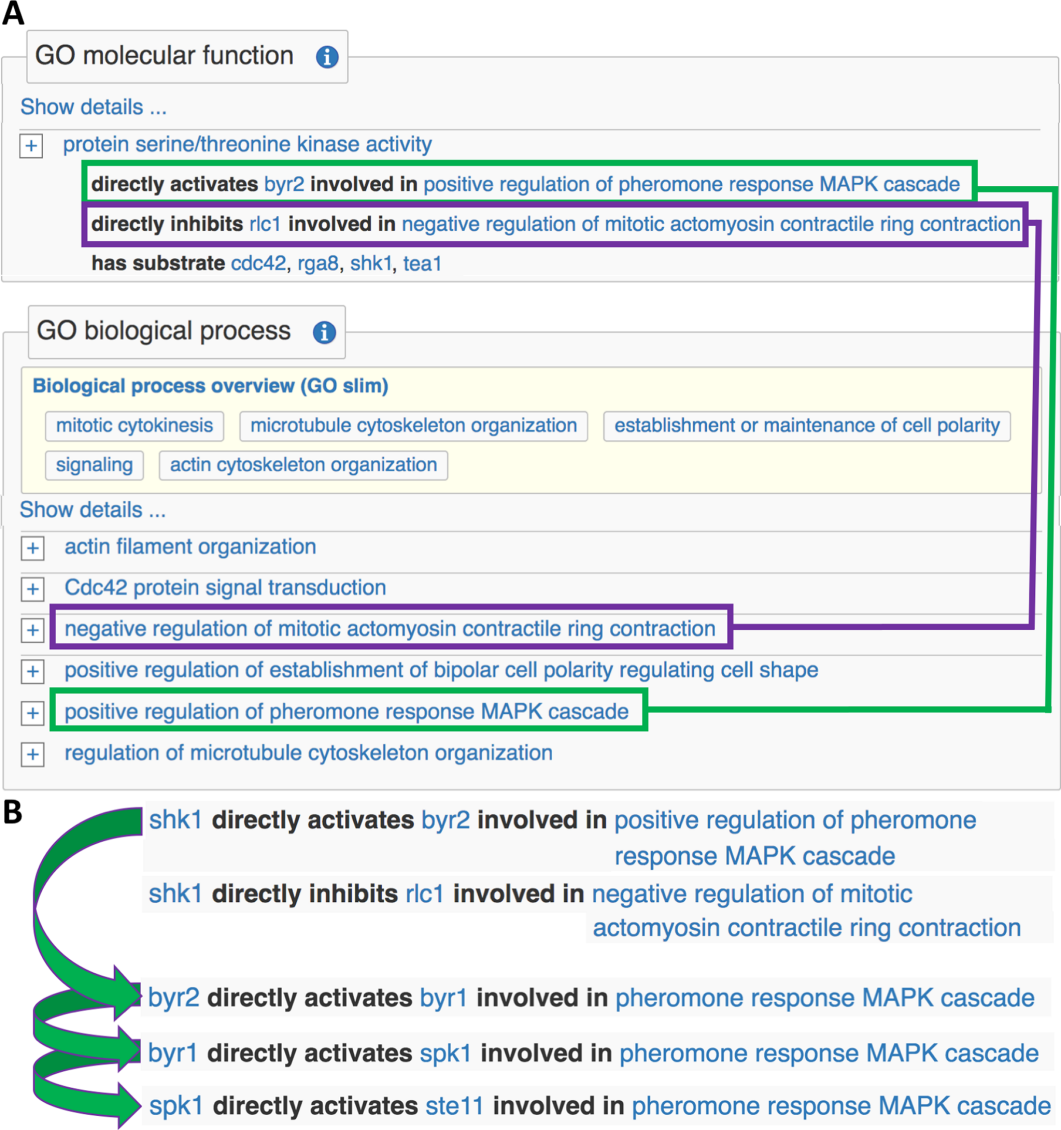
Linking GO molecular functions to substrates and GO biological processes using annotation extensions. (**A**) Examples from the *shk1* gene page illustrate uses of annotation extensions. In the GO molecular function section, an annotation indicates that Shk1 is a protein Ser/Thr kinase and extensions identify several of its substrates. Annotations in the GO biological process section show that Shk1 regulates several different pathways, but do not capture which phosphorylated substrates are relevant to which pathways. Extensions on the protein kinase molecular function annotation connect different substrates to specific processes (the underlying relation is *part_of*, converted to ‘involved in’ for a more human-friendly display). For example, Shk1 phosphorylates Byr1 as part of positive regulation of the pheromone MAPK cascade (green boxes). In another context, Shk1 phosphorylates Rlc1, Rlc1 is inhibited and contractile ring contraction is negatively regulated (purple boxes). (**B**) A representation of a signaling pathway can be built up from a series of molecular function annotations that use extensions to identify regulated kinase substrates and the biological process context in which the functions take place.

For an overview of fission yeast biology, PomBase provides a view based on a ‘GO slim’ (https://www.pombase.org/browse-curation/fission-yeast-go-slim-terms), comprising 53 GO biological process terms specific enough to be informative about a gene product's cellular role. This browsable resource represents 99.5% of all proteins that have a biological process annotation. The GO slim view links to term pages that display genes annotated to the term, grouped according to any more specific *child* term annotation. Using annotation extensions between molecular functions and biological processes as described below, each GO slim term connects to a curated network view derived from GO annotation extensions, displayed in esyN (12). The esyN network is supplemented by complex annotations (using GO cellular component terms) and direct physical interactions. GO slim terms associated with specific genes are also displayed on gene pages, at the top of the GO biological process section (Figure 1A).

### From annotations to networks

We have previously described (7) PomBase’s use of GO annotation extensions (13) to add specificity to functional annotation. More recently, we have expanded extension usage to link molecular functions to biological processes. As shown in Figure 1A, molecular function annotations can have extensions that link to biological processes via a *part_of* relation, displayed as ‘involved in’. Because one annotation connects an enzyme with both a substrate and a pathway, the annotations can be used to navigate a pathway from gene to gene, even when one enzyme acts on several substrates to regulate distinct pathways (Figure 1B). Conversely, the ‘target of’ section can be used to navigate pathways in the opposite direction.

### Making biological knowledge findable

#### New page types

To complement the gene-centric view of data on traditional gene pages, PomBase has added three new page types: for ontology terms, publications and genotypes.

**Figure 2.**
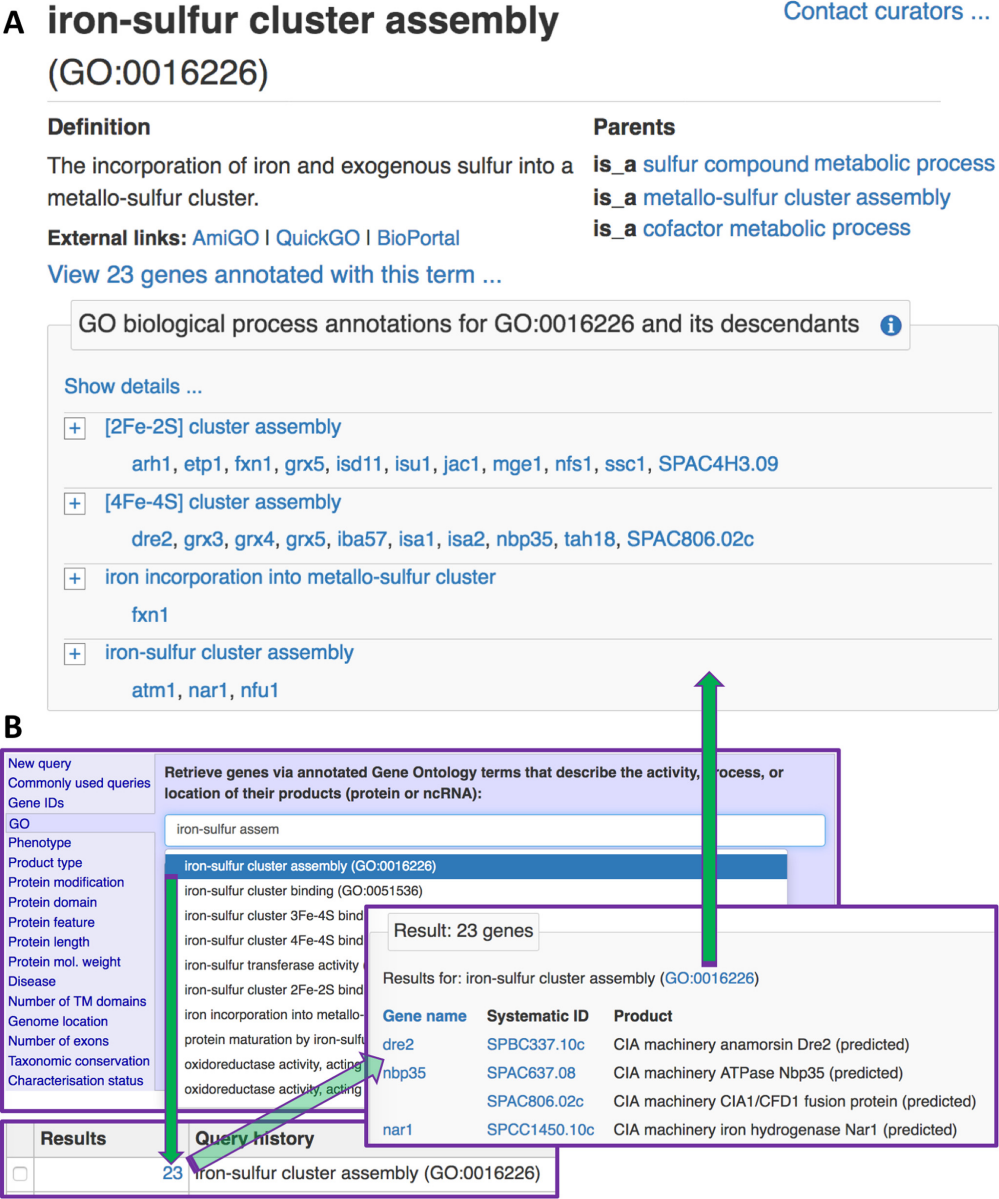
Using and finding ontology term pages. (**A**) Ontology term page layout. The page includes term details (name, ID, definition), links to external browsers and links to PomBase pages for more general terms in the ontology. Where applicable, annotations to the term are shown in a table, with summary and detailed views as on gene pages. Term pages use ontology structure to provide the ‘parent’ links and to distinguish annotations made directly to the term from annotation to any child terms. (**B**) Finding term pages. The PomBase advanced search can be used to find GO, phenotype (FYPO), protein modification and disease terms. On the search results page, a hyperlink goes to the term page. Term pages can also be found by searching for a term ID using the simple search box at the top of PomBase pages, or by following links where ontology terms appear in annotation extensions.

Ontology term pages were developed by greatly enhancing the pages listing genes annotated to specific ontology terms. The new term pages support improved browsing of genes, annotations and ontology structure; they are available for every ontology term used in annotations and in extensions. All term pages include the term definition, links to more general *parent* terms in the ontology, and links to external browsers. For GO, phenotypes and modifications, the pages display annotations and link to lists of annotated genes, which (in turn) can send the gene list, or a subset of the gene list, to the Advanced Search. As exemplified in Figure 2A, the term page display distinguishes annotations associated with more specific *child* terms from those connected directly to the viewed term, where applicable.

Publication pages list all annotations in PomBase that cite the paper, along with citation details, the abstract and links to PubMed and EuropePMC.

Genotype pages provide details of the alleles that make up each genotype, and list all associated phenotypes.

Ontology term, publication and genotype pages can be reached via hyperlinks on gene pages. Term pages can also be retrieved by querying by name or ID in the simple search (box in every page header) or in the Advanced Search (Figure 2B) The simple search can also be used to reach publication pages by searching for a PubMed ID, publication title or author name.

### Non-redundant data display

#### Display options

**Figure 3.**
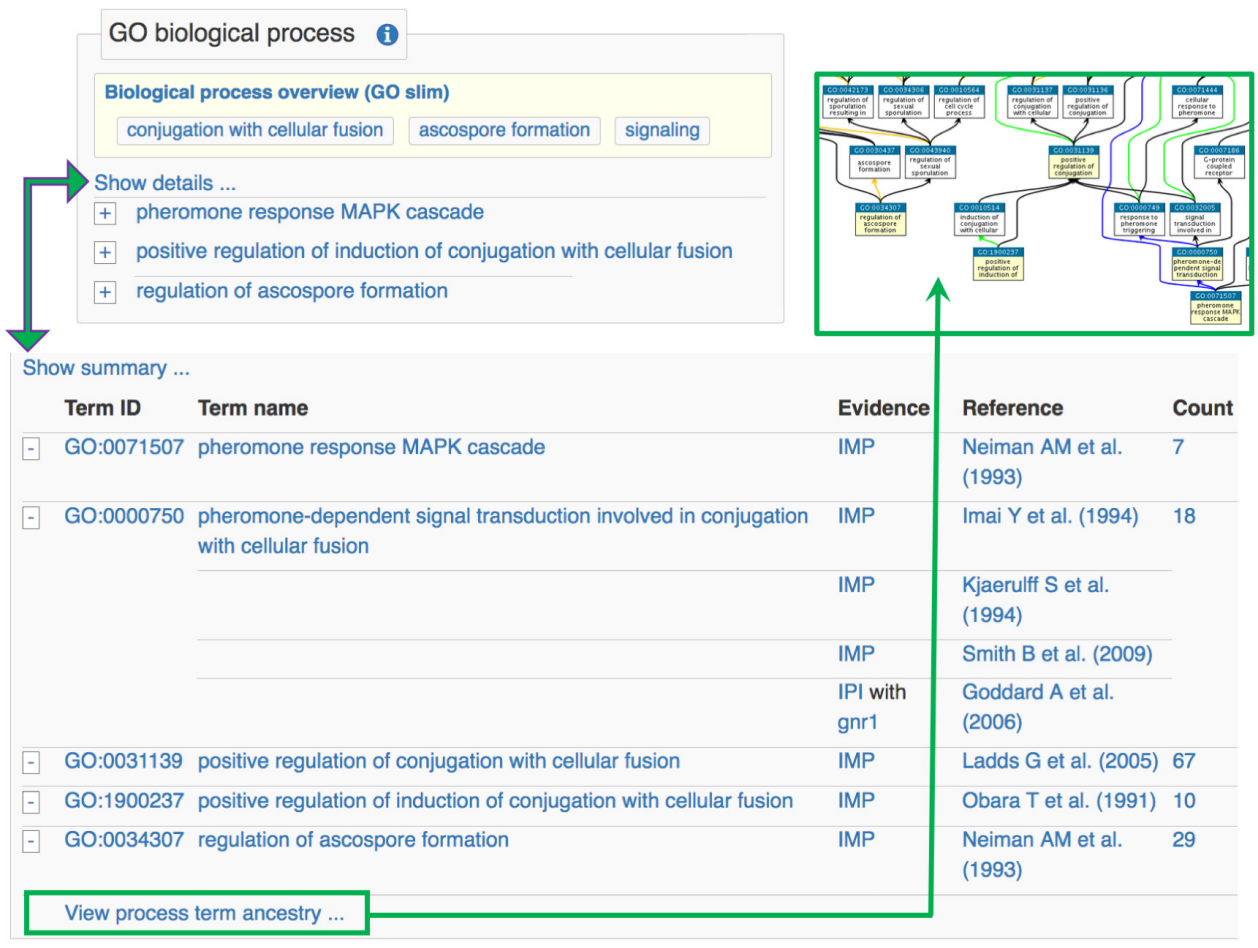
Annotation views. Tables of ontology-based annotations have simplified, non-redundant summary views (top) and comprehensive views showing details (bottom). ‘Show details’ and ‘Show summary’ links toggle between the views for all annotations in the table, and ± buttons switch views for individual annotations. For GO biological process terms, the detailed view also includes a ‘view ancestry’ link to a graphical ontology view (derived from QuickGO (14)) that highlights directly annotated terms and shows their links to more general terms (green boxes).

All pages that display ontology-based annotations offer two display options. By default, a summary display provides a concise view of the most specific annotations, taking ontology terms and extensions into account. Multiple manual annotations to a single term derived from different literature sources are allowed—as they represent reproduced results that increase confidence—but are hidden in the summary view. In the detailed view, these additional annotations are shown, as are annotations to less-specific terms that are included in the dataset to ensure comprehensive coverage of both historical and current literature. The detailed view also shows supporting metadata, such as evidence and references, and in the biological process section there is a link to view ontological relationships between processes (14). The detailed view can be enabled for a single annotation in the summary view, or for all annotations in a page section. Figure 3 shows the summary and detailed views of GO annotations on a gene page.

#### Phenotype display filtering

Phenotype annotations pose a particular usability challenge in PomBase, because pages for intensively studied genes may now display hundreds of unique phenotypes. To alleviate the burden of browsing enormous annotation tables, the display can now be filtered based on broad phenotypic categories such as cell viability, sensitivity to chemical, morphology or cell-cycle phenotypes. The detailed view can also be filtered on evidence, to show only phenotypes detected by specified methods, such as microscopy or cell growth assays.

#### External annotations and filtering

PomBase also incorporates annotations from external sources, notably interactions from BioGRID (15) and GO annotations from InterPro (16) and UniProtKB (17). Most of the externally sourced GO annotations are created by computational—rather than manual—methods, such as transfer of experimentally supported annotations from one species to an ortholog in another (see refs. (16,18). To avoid redundancy in the fission yeast GO annotation set, GO data are filtered prior to loading to remove computationally derived annotations that are identical to, or less-specific than, manually curated data. PomBase curators have refined filter parameters over time to optimize for broad coverage without excessive redundancy, and have begun to share the filtering approach with the rest of the GO Consortium.

#### JBrowse

PomBase now uses a JBrowse (19) instance as its genome browser (https://www.pombase.org/jbrowse/). All datasets from the old Ensembl-style browser have been incorporated, as well as newly published datasets (20–23, BioRxiv: https://doi.org/10.1101/281642) and genome feature tracks generated in-house. We have also improved the metadata associated with each track, capturing a broad and consistent range of experimental details to make datasets more findable. For example, tracks can be filtered by publication, by data type, for data generated in a specific mutant, or for data collected during a specific growth phase or during a stress response. Publication pages for papers describing browser datasets have links to JBrowse, with the relevant data track(s) enabled.

## CURATION AND THE POMBASE COMMUNITY

### Curation statistics

Curation statistics are now available on a web page, https://curation.pombase.org/pombe/stats/annotation, reachable via the ‘Genome status’ menu. This page quantifies a broad range of curation activities and is updated in parallel with data on PomBase pages. As of 30 August 2018, 56% of the curatable literature has been curated, yielding 235 045 manual annotations of assorted types. The statistics page includes graphs and links to data tables, breaking down annotations by type, and publications by curation status, over time.

### Outreach and promotion of fission yeast research

**Figure 4.**
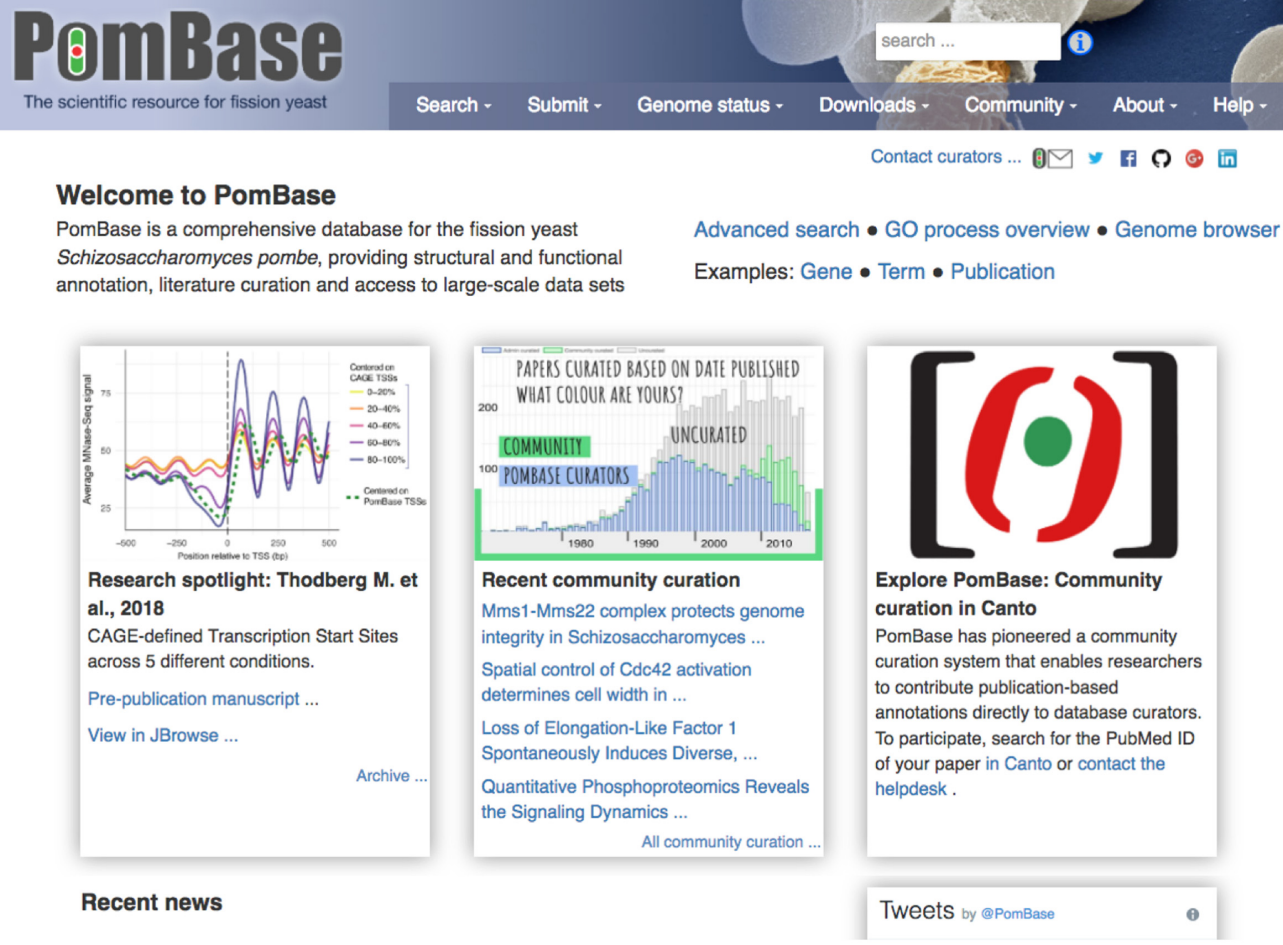
The new PomBase front page. The main panels highlight recent fission yeast research, community curation and PomBase usage tips (left, center and right, respectively). The page also provides news and links to example pages and frequently used tools.

The PomBase home page (Figure 4) has been reorganized to feature fission yeast research activities prominently, as well as to support convenient navigation around the site. Graphical abstracts of newly published and curated fission yeast literature are rotated in a ‘research spotlight’ panel, and another panel lists the publications most recently curated with community contributions. Both panels provide links to PomBase publication pages.

Community curation remains one of PomBase’s signature achievements, and has matured into a reliable system in which researchers and professional curators collaborate to create biologically accurate and computationally robust representations of experimental data. To date (30 August 2018) community curators have provided 11 199 annotations from 598 low-throughput publications, reflecting a response rate of [43.1%. Community contributions are acknowledged on PomBase publication pages.

## INFRASTRUCTURE AND IMPLEMENTATION

The new PomBase uses a suite of open-source tools and code to support frequent data updates and a responsive, intuitive user interface. The resulting system is modular, customizable and compatible with the Canto community annotation tool (24), and therefore readily reusable with data from other species.

### Chado, nightly updates and data export

Curation data is integrated nightly (GMT) into a PostgreSQL Chado (25) database, which is published on the PomBase FTP site (ftp://ftp.pombase.org/nightly_update/). The loading scripts implement annotation filtering using criteria established by curators as described above.

Data updated daily include:

- *Schizosaccharomyces pombe* community and professional curation from Canto
- PomBase sequence annotation
- Source ontologies
- Filtered data from external sources, including UniProt-GOA annotation (26) and BioGRID interactions (15) for *S. pombe*

Data is extracted from the Chado database into a set of intermediate files (JSON, GFF3 and FASTA). Nightly data updates draw from these files to populate PomBase pages and tools, including gene, term and reference pages, files downloadable from the FTP site, the advanced search feature, the JBrowse genome browser and the Solr/Lucene search engine.

The files, data and executables used to run the PomBase website are built into a Docker container and copied to a staging server each night. Upon passing automated checks, the container is copied to the main PomBase web server. Because the container holds a full instance of the website, it can be copied to development machines for troubleshooting should any checks fail.

### Docker container and server operation

The PomBase website is hosted on small Linux Virtual Machine running a single copy of the Docker container. Two processes run inside the container. The first (‘pombase-server’; https://github.com/pombase/pombase-chado-json) is implemented in Rust (https://www.rust-lang.org) and serves all JSON files and responds to advanced search queries. The second process is a Solr instance used for ontology term name autocompletion in the advanced search interface and for other text searching tasks.

The pombase-server process reads all the JSON files at start-up and holds everything in memory, including all the data needed for the query builder. This model of a single-process with no backing database makes serving pages and answering advanced search queries very fast. The pombase-server process also handles term name autocompletion by proxying requests from the client to the Solr server instance.

### Client side code

The client-side code for PomBase has been re-written as a ‘Single Page Application’ using the Angular framework (https://angular.io/) to improve speed and responsiveness. As the user moves around the site the client makes HTTP/2 requests to the server to fetch JSON documents corresponding to each section of the site. These requests are cached in the browser, making the site highly responsive to user interaction.

## FUTURE DIRECTIONS

We will continue to provide comprehensive literature curation, increase participation in community curation, and incorporate submitted large-scale datasets into PomBase JBrowse. We are evaluating novel ways to display information on gene pages, such as interactive displays for networks and pathways, and graphical displays of protein modifications and mutations. We will also pursue ways to build the growing body of interconnected functional annotations into models that represent all pathways in a unicellular eukaryotic cell. For phenotype data, both querying and filtering will be extended to use alleles and experimental conditions as options. Our ongoing efforts will improve not only PomBase's own data acquisition and use, but also its interoperability with other fungal informatics resources, enabling numerous databases to make optimal use of available information.
